# Lactobacillus Bacteremia Following Dental Instrumentation in a Patient With a Bioprosthetic Valve

**DOI:** 10.7759/cureus.89558

**Published:** 2025-08-07

**Authors:** Agnieszka Gryguc-Saxanoff

**Affiliations:** 1 Internal Medicine, Ronald Reagan University of California Los Angeles Medical Center, Los Angeles, USA

**Keywords:** anaerobic gram-positive rod, bentall procedure, lactobacillus bacteremia, prosthetic heart valve, valvular endocarditis

## Abstract

Although generally considered harmless commensals or beneficial probiotics, *Lactobacillus* species can act as opportunistic pathogens under certain clinical conditions. We describe a case of high-grade *Lactobacillus* bacteremia in a 59-year-old man with a history of aortic root dilation status post Bentall procedure and bioprosthetic aortic valve replacement. The suspected source was recent dental instrumentation. Despite negative imaging for vegetations, the patient was treated empirically for prosthetic valve endocarditis with six weeks of intravenous penicillin. This case reminds clinicians to take *Lactobacillus* bacteremia seriously in the proper clinical context, especially when prosthetic material is present.

## Introduction

*Lactobacillus* species are gram-positive, facultatively anaerobic rods commonly found in the gastrointestinal tract, genitourinary tract, oropharynx, oral cavity, dental plaque, and female reproductive tract. They are generally regarded as beneficial commensals and are widely included in probiotic supplements. However, under certain conditions, they can act as opportunistic pathogens. Emerging literature has raised concerns about potential risks associated with probiotic use in vulnerable populations, including individuals with structural heart disease, prosthetic material, or compromised mucosal barriers. In such patients, the isolation of *Lactobacillus* from blood cultures, regardless of probiotic use, may indicate actual infection rather than contamination. Although rare, cases of *Lactobacillus* bacteremia and endocarditis are increasingly documented. In a review of more than 200 cases, nearly one-third represented confirmed infections, many involving endocarditis [1]. A national surveillance study from Finland also observed a temporal association between increased probiotic use and higher rates of *Lactobacillus* bacteremia, although a causal link was not definitively established [2].

## Case presentation

A 59-year-old man with a history of aortic root dilation who had undergone a composite aortic root replacement (Bentall procedure) with bioprosthetic aortic valve replacement and coronary artery bypass surgery in September 2024 presented in early June 2025 with fatigue, malaise, and chills. He had been undergoing dental procedures since December 2024, including cavity repairs, crown placement, and bone grafting. Outpatient blood cultures drawn on June 4 grew high-grade *Lactobacillus* in multiple bottles. He was referred to the hospital and admitted on June 7. Repeat admission blood cultures were also positive for *Lactobacillus*, though subsequent cultures were negative. A transesophageal echocardiogram performed on June 9 showed no vegetations or evidence of prosthetic valve dysfunction. Given the high-grade bacteremia and the presence of a bioprosthetic aortic valve, empiric treatment for prosthetic valve endocarditis was initiated. He received a continuous infusion of intravenous penicillin G, with a planned six-week course that was to continue through July 20. The patient was discharged in stable condition and did well at home. On July 9, he returned with a low-grade fever (100.4°F) and chills. He was tachycardic and had a white blood cell count of 14,000 cells per microliter. Imaging studies and repeat blood cultures were unremarkable. A repeat transesophageal echocardiogram again showed no evidence of endocarditis. Broad-spectrum antibiotics were discontinued, and he was resumed on penicillin to complete the original treatment course. During this admission, the patient's sister shared that two family members had been diagnosed with familial Mediterranean fever through private genetic testing. Due to the patient's history of vague, recurring symptoms, outpatient testing for mutations in the Mediterranean fever gene was arranged.

Lab trends

White blood cell count peaked at 14.18 and trended down to 9.60 over three days (Table 1). Inflammatory markers were decreased over time. C-reactive protein decreased from 6.3 mg/dL to 1.5 mg/dL (Table 2). Blood cultures on 6/4 and 6/6 grew *Lactobacillus *sensitive to penicillin; all subsequent cultures were negative (Table 3).

**Table 1:** White blood cell trend

Date	Day	WBC (×10⁹/L)
July 9, 2025	Day 1	14.18
July 10, 2025	Day 2	11.20
July 11, 2025	Day 3	9.60

**Table 2:** Inflammatory marker trends CRP: C-reactive protein.

Date	CRP (mg/dL)	Procalcitonin (ng/mL)
June 4, 2025	6.3	0.1
June 6, 2025	3.3	<0.10
June 26, 2025	1.5	<0.10
July 9, 2025	Not measured	0.18

**Table 3:** Lactobacillus susceptibility report

Antibiotic	MIC (mcg/mL)	Interpretation
Daptomycin	2	Susceptible
Penicillin	1	Susceptible

## Discussion

*Lactobacillus *species, although widely regarded as non-pathogenic commensals or probiotic organisms, can act as opportunistic pathogens in select patient populations. Severe infections caused by *Lactobacillus*, including bacteremia, endocarditis, liver abscesses, and septic arthritis, have been documented, particularly in individuals with prosthetic devices, mucosal disruption, or immunocompromising conditions [3,4]. In patients with prosthetic heart valves and a history of recent dental procedures, the presence of *Lactobacillus* in blood cultures should prompt a careful evaluation, as it may represent an actual infection rather than incidental contamination. In the clinical scenario described above, the investigation included two transesophageal echocardiograms (TEEs), which failed to reveal vegetations. It is essential to note that imaging in the context of prosthetic valves is known to be limited. Due to acoustic shadowing and atypical vegetations, this often leads to false-negative studies. As noted in contemporary reviews, clinical decision-making in suspected prosthetic valve endocarditis typically relies on microbiologic and clinical findings, often in the absence of definitive imaging [5]. The pathogenicity of *Lactobacillus* may be underestimated. Some strains express surface adhesion molecules, including sortase-anchored pili, S-layer proteins, and multifunctional enzymes, that facilitate adherence to mucin, fibronectin, and collagen, particularly on damaged endothelium or prosthetic surfaces [6-8]. Additionally, the ability of certain strains to form biofilms increases their potential for persistent infection [6]. From a therapeutic standpoint, antimicrobial resistance is an important consideration. Many *Lactobacillus* species exhibit intrinsic resistance to vancomycin, which is often part of empiric endocarditis regimens. Fortunately, most isolates remain susceptible to penicillin and other beta-lactams [9]. Prolonged intravenous beta-lactam therapy, often for four to six weeks, is supported by case reports and narrative reviews. Some clinicians advocate for combination therapy with aminoglycosides in select cases, though high-quality evidence is limited [9-11]. The role of probiotics in invasive *Lactobacillus* infections has also been explored. A national surveillance study in Finland observed a temporal increase in *Lactobacillus rhamnosus* GG bacteremia that paralleled the rising use of probiotics. Although a causal relationship was not definitively established, the findings raised concerns about the safety of probiotics in high-risk individuals [2]. Other reports have described bacteremia in critically ill patients following probiotic administration, especially in those with central lines or compromised mucosal barriers [6,11]. This case also raises the possibility of an alternative or concurrent diagnosis. Familial Mediterranean fever, a hereditary autoinflammatory condition, can present with intermittent fevers and elevated inflammatory markers, which can mimic an infection. Although typically diagnosed earlier in life, adult-onset or mild phenotypes are well described. In patients with nonspecific systemic symptoms and a family history suggestive of FMF, genetic testing should be considered.

## Conclusions

This case underscores the importance of recognizing *Lactobacillus *as a potential pathogen in the appropriate clinical context. In patients with prosthetic valves and recent dental manipulation, even the absence of confirmatory imaging should not preclude treatment when the clinical picture is concerning. Additionally, this case illustrates the diagnostic complexity that arises when symptoms of infection overlap with non-infectious etiologies. Familial Mediterranean fever, an autoinflammatory condition, can present with intermittent fevers and elevated inflammatory markers, potentially mimicking infectious endocarditis. Ultimately, managing such cases requires not just guidelines, but careful clinical judgment, a willingness to think broadly, and attention to the whole story the patient presents.
